# Additions and corrections to the check list of the Noctuoidea (Insecta, Lepidoptera) of North America north of Mexico

**DOI:** 10.3897/zookeys.264.4443

**Published:** 2013-02-06

**Authors:** J. Donald Lafontaine, B. Christian Schmidt

**Affiliations:** 1 Canadian National Collection of Insects, Arachnids, and Nematodes, Biodiversity Program, Agriculture and Agri-Food Canada, K.W. Neatby Bldg., 960 Carling Ave., Ottawa, Ontario, Canada K1A 0C6; 2 Canadian Food Inspection Agency, Canadian National Collection of Insects, Arachnids, and Nematodes, K.W. Neatby Bldg., 960 Carling Ave., Ottawa, Ontario, Canada K1A 0C6

**Keywords:** Canada, United States, Erebidae, Noctuidae, Nolidae, Boletobiinae, Eligminae, Diphtherinae, Eulepidotinae, Omopterinae, Toxocampinae, Boletobiini, Aventiini, Eublemmini, Phytometrini

## Abstract

A total of 64 additions and corrections are listed and discussed for the check list of the Noctuoidea of North America north of Mexico published in 2010. One family-group name is inserted, four are changed in rank, one is deleted, one is changed in name, and three are changed in authorship. Taxonomic changes to species are six new or revised synonymies, one new combination, and one revision in status from species to subspecies.

## Introduction

Continuing work on the taxonomy and systematics of New World Noctuoidea has resulted in an additional 64 changes to the check list North American Noctuoidea (Lafontaine and Schmidt 2010), these in addition to the 96 changes published in 2011 (Lafontaine and Schmidt 2011). Eighteen species are added to the fauna, eight are removed through synonymy, and eight are name changes due to synonymy. The new total for Noctuoidea in North America north of Mexico is 3689.

## Materials and methods

### Repository abbreviations

Taxonomic changes are based on examination of material, especially type specimens, in the following collections:

**BMNH** The Natural History Museum [statutorialy: British Museum (Natural History)], London, UK

**CNC** Canadian National Collection of Insects, Arachnids, and Nematodes, Ottawa, Ontario, Canada

**DFC** David Fine Collection, Coconut Creek, Florida, USA

**JKAC** James K. Adams Collection, Calhoun, Georgia, USA

**JTTC** James T. Troubridge Collection, Selkirk, Ontario, Canada

**MNHN** Muséum National d’ Histoire Naturelle, Paris, France

**TLSRC** Texas Lepidoptera Survey Research Collection, Houston, Texas, USA

**TSDC** Terhune S. Dickel Collection, Anthony, Florida, USA

**USNM** National Museum of Natural History [formerly, United States National Museum], Washington, District of Columbia, USA

## Results

**Corrections, additions, and changes** (highlighted in **bold**)

p. 3 **Tribe Boletobiini** [insert after Subfamily Boletobiinae]

p. 3 **Subfamily Eligminae Mell, 1943** [insert before subfamily Risobinae]

p. 3 **Subfamily Diphtherinae** [insert before subfamily Nolinae]

p. 3 & p. 28 **Tribe Aventiini** [lower subfamily name to tribal name]

p. 3 & p. 28 **Tribe Eublemmini** [lower subfamily name to tribal name]

p. 3 & p. 29 **Tribe Phytometrini** [lower subfamily name to tribal name]

p. 3 & p. 30 **Subfamily Toxocampinae** [raise tribal name to subfamily name]

p. 3 & p. 37 **Tribe Omopterini Boisduval, 1833** [change Tribe Ophiusini Guenée, 1837 to Omopterini]

p. 3 & p. 39 **Tribe Eulepidotini** and **Tribe Panopodini** [delete tribal names]

p. 3 & p. 42 Collomeninae **Zahiri, Lafontaine, & Schmidt, 2012** [correct authorship from Kitching and Rawlins [1998]]

p. 3 & p. 49 **Subfamily Raphiinae Beck, 1996 [**change Subfamily Dilobinae to Subfamily Raphiinae]

p. 4 & p. 50 Acronictinae **Harris, 1841** [correct authorship from Heinemann, 1859]

p. 5 & p. 99 Agrotina **Harris, 1841** [correct authorship from Rambur, 1848]

p. 28 **Tribe Boletobiini** [insert to include species 930673 to 930692]

p. 41 **Subfamily Diphtherinae and 931410 *Diphthera festiva* (Fabricius, 1775)** [insert before Subfamily Nolinae and renumber species as **931120.1**]

p. 42 **Subfamily Eligminae Mell, 1943** [insert before 931147 *Iscadia aperta* Walker]

p. 49 **Delete** Subfamily Diphtherinae and 931410 *Diphthera festiva* (Fabricius, 1775)

**930356.1 *Xenosoma flaviceps* (Walker, 1865)**

930405 *Cycnia oregonensis* (Stretch [1874])

** ssp. *Cycnia oregonensis oregonensis* (Stretch [1874])**

** ssp. *Cycnia oregonensis tristis* Crabo, 2013**

**930447.1 *Aclytia heber* (Cramer, [1780])**

930500 *Zanclognatha jacchusalis* (Walker, 1859)

** ssp. *Zanclognatha jacchusalis jacchusalis* (Walker, 1859)**

 syn. *Zanclognatha ochreipennis* (Grote, 1872)

** ssp. *Zanclognatha jacchusalis lutalba* (Smith, 1906)**

** ssp. *Zanclognatha jacchusalis bryanti* Barnes, 1928**

**930501 Delete.** Moved to synonymy of 930500

930502 *Chytolita morbidalis* (Guenée, 1854)

** syn. *Chytolita petrealis* Grote, 1880**

** syn. *Chytolita punctiformis* (Smith, 1895)**

** syn. *Chytolita fulicalis* Smith, 1907**

**930503 Delete.** Moved to synonymy of **93**0502

930579***Hypena lividalis* (Hübner, 1796)**

** syn. *Hypena abjuralis* Walker, [1859]**

930608 *Anomis illita* Guenée, 1852

** syn. *Anomis conducta* Walker, [1858]**

** syn. *Anomis hostia* (Harvey, 1876)**

930609 ***Anomis gentilis* Schaus, 1912**

** syn. *Anomis exacta* of authors, not Hübner, 1822**

**930611.1 *Dinumma deponens* Walker, 1858**

**930621.1 *Gonodontodes dispar* Hampson, 1913**

**930702 Delete.** Moved to synonymy of 930705

930705 *Hemeroplanis historialis* (Grote, 1882)

** syn. *Hemeroplanis finitima* (Smith, 1893)**

** syn. *Hemeroplanis secundalis* (Smith, 1907)**

**930706 Delete.** Moved to synonymy of 930705

**930755.1 *Hemeroblemma mexicana* (Guenée, 1852)**

**930884 *Forsebia cinis* (Guenée, 1852)**

** syn. *Forsebia perlaeta* (H. Edwards, 1882)**

**930903.1 *Drasteria parallela* Crabo & Mustelin, 2013**

**930968.1** *Ophisma tropicalis* (Guenée, 1852)

**930968.2** *Mimophisma delunaris* (Guenée, 1852)

**930968.3** *Achaea ablunaris* (Guenée, 1852)

**931057Delete.** Moved to **930968.1**

**931058 Delete.** Moved to **930968.2**

**931059 Delete.** Moved to **930968.3**

**931109 Delete.** Moved to synonymy of 931117

**931113.1 *Paectes fuscescens* (Walker, 1855)**

**931116 Delete.** North American concept moved to synonymy of 931117.1

**931117 Delete.** North American concept moved to synonymy of 931117

**931117 *Paectes nana* (Walker, 1865)**

** syn. *Paectes arcigera* of authors, not (Guenée, 1852)**

** syn. *Paectes burserae* (Dyar, 1901)**

**931117.1 *Paectes asper* Pogue, 2013**

**syn. *Paectes obrotunda* of authors, not (Guenée, 1852)**

**931167.1 *Enigmogramma antillea* Becker, 2001**

**931171.1 *Chrysodeixis chalcites*** (Esper, 1789)

**syn. *Chrysodeixis eriosoma* of Lafontaine & Schmidt (2010), not (Doubleday, 1843)**

**931253.1 *Amyna amplificans* (Walker, 1858)**

**931260.1 *Tripudia paraplesia* Pogue, 2009**

**931931 *Sympistis zetterstedtii* (Staudinger, 1857)**

 ssp. *Sympistis zetterstedtii kolthoffi* (Aurivillius, 1890)

**931965 *Eudryas brevipennis bonneville* Shepard & Crabo, 2013**

**931988.1 *Perigea bahamica* Hampson, 1908**

**932006 *Condica charada* (Schaus, 1906)**

**932021.1 *Ogdoconta satana* Metzler, Knudson & Poole, 2013**

**932023 *Ogdoconta rufipenna* Metzler, Knudson & Poole, 2013**

 syn. *Ogdoconta* sp. not *Ogdoconta lilacina* (Druce, 1890) (Lafontaine and Schmidt 2010).

**932023.1 *Ogdoconta fergusoni* Metzler & Lafontaine, 2013**

**932061 *Protoschinia*** *nuchalis* (Grote, 1878)

**932368.1 *Resapamea diluvius* Crabo, 2013**

**932368.2 *Resapamea angelika* Crabo, 2013**

**932368.3 *Resapamea mammuthus* Crabo, 2013**

**932454 Delete.** Moved to synonymy of 932455

932455 *Hydraecia medialis* Smith, 1892

** syn. *Hydraecia pallescens* Smith, 1899**

932456 *Hydraecia obliqua* **(Harvey)**, 1876

** syn. *Hydraecia ximena* (Barnes & Benjamin, 1924)**

** syn. *Hydraecia columbia* (Barnes & Benjamin, 1924)**

**932462 Delete.** Moved to synonymy of 932456

**932463 Delete.** Moved to synonymy of 932456

932631 *Aseptis* ***fanatica*** Mustelin, 2006

**932693.1 *Fishia nigrescens* Hammond & Crabo, 2013**

**932711.1 *Ufeus felsensteini* Lafontaine & Walsh, 2013**

**933606 *Xestia perquiritata orca* Crabo & Hammond, 2013**

## Notes

**p. 3 & p. 28–29 subfamily Boletobiinae –** The phylogenetic studies of the Erebidae by Zahiri et al. (2012a) showed that the subfamilies Aventiinae, Eublemminae, and Phytometrinae should be included within the subfamily Boletobiinae as tribes.

**p. 3 & p. 30 Subfamily Toxocampinae –** phylogenetic studies of the Erebidae by Zahiri et al. (2012a) showed that the Toxocampinishould be treated as a subfamily of the Erebidae, rather than as a tribe of the Erebinae.

**p. 3 & p. 37 Tribe Omopterini –** phylogenetic studies of the Erebidae by Zahiri et al. (2012a) showed that the tribe Ophiusini Guenée is confined to the Old World, so the tribal name for most of the New World representatives related to *Zale* Hübner should be changed to tribe Omopterini. Three genera (*Ophisma* Guenée, *Mimophisma* Hampson, *Achaea* Hübner) should be transferred to the Poaphilini.

**p. 3 & p. 39 Subfamily Eulepidotinae –** The nuclear DNA results in Zahiri et al. (2012a) showed that genera *Eulepidotis* and *Panopoda* are closely related, so the tribes Eulepidotini and Panopodini are unnecessary.The date of the Eulepidotinae was corrected from 1985 to 1895 by Lafontaine and Schmidt (2011).

**p. 3 & p. 41 Subfamily Diphtherinae –** The molecular results of Zahiri et al. (2012b) show that the subfamily Diphtherinae is the basal lineage of the Nolidae and so it is moved to a position as the first subfamily of the Nolidae. These results show very strong support for the monophyly of the subfamilies of the Nolidae but virtually no support for phylogenetic associations among the subfamilies, other than Diphtherinae being sister to the other subfamilies. So, we suggest no change in subfamily sequence at this time.

**p. 3 & p. 42 Subfamily Eligminae –** The molecular results of Zahiri et al. (2012b) showed that the genus *Iscadia* Walker, and related Neotropical genera (e.g., *Elaeognatha* Hampson) belong to the Eligminae and not to the Chloephorinae: Sarrothropini as previously supposed. Previous to these results the Eligminae were thought to be restricted to Asia and Australia.

**p. 3 & p. 49 Subfamily Raphiinae –** Change subfamily name from Dilobinae (see Lafontaine and Schmidt 2013).

**p. 4 & p. 50 Acronictinae –** Change in authorship from Schmidt and Lafontaine 2013.

**p. 5 & p. 99 Agrotina –** Change in authorship from Schmidt and Lafontaine 2013.

**p. 42 Subfamily Collomeninae –** The subfamily name was first used in a North American check list (Franclemont and Todd 1983), but without a description. This was discussed in more detail by Kitching and Rawlins [1998], but mostly from a distributional perspective and still no diagnostic characters were given that would validate the subfamily name by the rules of the International Commission on Zoological Nomenclature. A formal description was given by Zahiri, Lafontaine and Schmidt (2012c).

**p. 49 Subfamily Diphtherinae –** Moved to Nolidae.

**930356.1 *Xenosoma flaviceps*–** This species occurs in northern Mexico (State of San Luis Potosi), and in southern Mexico from at least Chiapas to Guatemala and Costa Rica. A single specimen was collected at Alamo, Texas on 5 December 2012. Contributed by E. Knudson and C. Bordelon. Voucher in TLSRC, photograph examined.

**930405 *Cycnia oregonensis tristis* –** New subspecies (see Crabo et al. 2013).

**930447.1 *Aclytia heber* –** A single specimen was collected at Alamo, Texas in November of 2012. Contributed by E. Knudson and C. Bordelon. Voucher in TLSRC, photograph examined.

**930500 *Zanclognatha jacchusalis*–** The species is widely distributed in eastern United States and occurs as far west as Arizona. It is characterized by the burnt-orange forewing ground color that is heavily speckled with black scales. It is replaced in Canada by a form that has paler buffy-brown or gray-brown forewings with little black speckling. This northern form is currently treated as *Zanclognatha lutalba* (Smith), occurring from Nova Scotia to Alberta, and as *Zanclognatha lutalba* ssp. *bryanti* Barnes in British Columbia and Washington. However, there is a broad area in southern Ontario and Quebec, northern New York, and New England, where most specimens are intermediate between typical *Zanclognatha jacchusalis* and *Zanclognatha lutalba* and occasionally *Zanclognatha lutalba*-like forms are found as far south as the Appalachians of North Carolina. There are no external structural or genital characters to distinguish the two taxa and barcodes do not separate them either, so we synonymize *Zanclognatha lutalba*, **syn. n.**, but retain the name as a northern subspecies as *Zanclognatha jaccusalis* ssp. *lutalba*, **stat. n.**, and move subspecies *bryanti* to *Zanclognatha jaccusalis* ssp. *bryanti*, **stat. rev.**

**930502 *Chytolita morbidalis*–** New and revised synonymy from Crabo et al. 2013.

**930579 *Hypena lividalis*–** The species, which occurs in Africa and southern Europe, is now believed to be a Pan-tropical species with *Hypena abjuralis* (Walker, [1859]), **syn. rev.**, as a synonym. The external characters, genitalia, and barcodes are the same from each region. It is possible that the spread of the species to the New World was aided by man.

**930608 *Anomis illita* –** Examination of type material and barcodes shows that *Anomis conducta* Walker, [1858], **syn. n.**, and *Aletia hostia* Harvey, 1876, **syn. n.**, are synonyms of *Anomis illita*, and not synonymsof *Anomis exacta* Hübner, 1822. The barcodes also show that *Anomis illita* is widely distributed from Florida and Texas southward through the Caribbean and Central and South America to Brazil, the latter being the type locality.

**930608 *Anomis gentilis* –** Examination of the type material associated with the name *Anomis exacta* and *Anomis gentilis* Schaus, shows that the species that occurs in Texas is *Anomis gentilis*. The type material of *Anomis exacta* is lost, but the illustration in Hübner (1822), and material associated with it in the Natural History Museum, London (BMNH), do not match the taxon occurring in Texas, or any North American species of *Anomis*. Further, the two names currently associated with the *Anomis exacta* as synonyms, one of which (*Anomis hostia* (Harvey)) was described from Texas, are synonyms of *Anomis illita* and are transferred to its synonymy above.

**930611.1 *Dinumma deponens* –** A fresh female** **specimen of *Dinumma deponens* Walker, 1858 was taken at gas station lights in Morganton, Fannin Co., in north Georgia on June 15, 2012 by Paul Dennehy and James Adams.  This location is quite rural, along a state highway about 100 miles north of Atlanta.  The species’ home range is “from India across E China to Japan, Korea and to Thailand” (Alberto Zilli, pers. comm.), though not Borneo.  The larval food plants of *Dinumma* Walker, and the species *Dinumma deponens*, are members of the genus *Albizia* Durazz. (Mimosa).  Mimosa is extensively planted and naturalized throughout north Georgia.  As such, it is certainly plausible that this single specimen represents a member of an established population of the moth in the U.S. It is tentatively included in the Anomini following Holloway (2005). The specimen is in the JKAC. Contributed by James Adams.

**930621.1 *Gonodontodes dispar*–** A male of this species was collected at Mile Marker 36, US A1A, Key Largo, Monroe Co., Florida, 2 May 2009 by David Fine. The specimen is in DFC. Contributed by Leroy Koehn.

**930705 *Hemeroplanis historialis* –** Examination of genitalia and barcodes shows that the names *Hemeroplanis finitima*, **syn. n.**, and *Hemeroplanis secundalis*, **syn. n.**, are color forms of *Hemeroplanis historialis*. The species is mainly distinguished from *Hemeroplanis incusalis*, which occurs with it in parts of Arizona and California, by the more parallel transverse lines on the forewing with black wedge-shaped spots on the costa in *Hemeroplanis historialis*, and usually the reniform spot is black.

**930755.1 *Hemeroblemma mexicana* –** A female of this species was collected 6 June 2012 at Falcon Heights, Starr Co., Texas by Barry Nall. The specimen is in the TLSRC.

**930884 *Forsebia cinis* –** The male lectotype of *Bolina cinis* Guenée, 1852, in the MNHN, Paris, is a senior synonym of *Forsebia perlaeta* (H. Edwards, 1882), **syn. n.**, and not a synonym of *Melipotis jucunda* Hübner, 1818, as previously supposed. *Forsebia cinis* is a **new combination.** Contributed by Robert Poole.

**930903.1 *Drasteria parallela* –** Addition (see Crabo et al. 2013).

**930961.1 *Ophisma tropicalis* –** The molecular results in Zahiri et al. (2012a) show that *Ophisma*, and the two genera below (*Mimophisma* Hampson and *Achaea* Hübner), should be in the Poaphilini, so the species is moved here from 931057.

**930961.2 *Mimophisma delunaris* –** moved from 931058.

**930961.3 *Achaea ablunaris* –** moved from 931059.

**931113.1 *Paectes fuscescens*–** A single specimen from Florida was found in unidentified material in the USNM by Mike Pogue while sorting specimens for a revision of the *Paectes arcigera* group. The specimen was reared but the data do not include host plant information. Terhune Dickel and Jim Troubridge have both collected this species in southern Florida (Homestead and Key Largo). Some specimens have previously been identified as *Paectes burserae*. Vouchers in CNC, JTTC, TSDC, and USNM.

**931117 *Paectes nana*–** This taxon, formerly considered to be a synonym of *Paectes arcigera*, is widely distributed in Florida, the Caribbean, Mexico, Central America, and northern South America. The taxonomic status and distribution are based on Pogue (2013). *Paectes arcigera* is found in the Lesser Antilles and Puerto Rico and would not be expected to occur in Florida. *Paectes burserae* (931109 in Lafontaine & Schmidt 2010) is placed in synonymy with *Paectes nana* by Pogue 2013.

**931117.1 *Paectes asper*–** Addition (see Pogue 2013). This is the species formerly identified as *Paectes obrotunda* (Guenée, 1852) in Florida. It is widely distributed in southern Florida and the Caribbean, whereas *Paectes obrotunda* is confined to Brazil (Pogue 2013).

**931167.1 *Enigmogramma antillea* –** This species was described by Becker (2001). A specimen was collected in Collier County, Florida, in 2012 by Jim Troubridge. Voucher in CNC.

**931171.1 *Chrysodeixis chalcites* –** This species occurs mainly in Africa and western Eurasia with *Chrysodeixis eriosoma* mainly in eastern Asia, but occasionally found in Europe in greenhouses. The two species are very difficult to identify other than by geographic range, DNA, and pheromones. When populations of this complex were discovered in greenhouses in British Columbia in 2006, it was assumed they would have an eastern Eurasian origin, like most recent introductions into the Vancouver area. More recently, barcode results show that these populations, and recently discovered populations in southern Ontario and Michigan, are referable *Chrysodeixis chalcites* and not to *Chrysodeixis eriosoma* (Murillo et al. in press).

**931253.1 *Amyna amplificans* –** A single specimen of this species was collectedby Bruce Walsh in the Huachuca Mountains in southeastern Arizona. Voucher in JBWC.

**931260.1 *Tripudia paraplesia* –** A single specimen of this species was collected by Vernon Brou in Louisiana in 1994. Previously, the species was recorded only as far north as northeastern Mexico (Pogue 2009). It is distinguishable from *Tripudia quadrifera* (Zeller) and *Tripudia rectangula* Pogue only by genital characters. Voucher in CNC.

**931931 *Sympistis zetterstedtii* –** Reinstated as a full species, not a subspecies of *Sympistis nigrita* (Boisduval, 1840) from the Alps, following Lafontaine and Schmidt (2013).

**931965 *Eudryas brevipennis bonneville* –** New subspecies (see Crabo et al. 2013).

**931988.1 *Perigea bahamica* –** The species was collected in Monroe County, Florida, in 2012 by Jim Troubridge. Voucher in CNC.

**932006 *Condica charada*** – The species name was misspelled as *chardra* in Lafontaine & Schmidt (2011).

**932021.1 *Ogdoconta satana* –** Addition (see Metzler et al. 2013).

**932023 *Ogdoconta rufipenna* –** Addition (see Metzler et al. 2013). This species had been listed in numerous lists and season summaries and was treated in Lafontaine & Schmidt (2010) as *Ogdoconta* sp. not *Ogdoconta lilacina* (Druce, 1890).

**932023.1 *Ogdoconta fergusoni* –** Addition (see Metzler et al. 2013).

**932061 *Protoschinia nuchalis* –** Change in combination from Lafontaine and Schmidt (2013).

**932368.1 *Resapamea diluvius* –** Addition (see Crabo et al. 2013).

**932368.2 *Resapamea angelika* –** Addition (see Crabo et al. 2013).

**932368.3 *Resapamea mammuthus* –** Addition (see Crabo et al. 2013).

**932455 *Hydraecia medialis* –**New synonymy from Crabo et al. 2013.

**932456 *Hydraecia obliqua* –** The author’s name, Harvey, should be in parentheses because the original combination was *Gortyna obliqua* Harvey. New synonymy from Crabo et al. 2013.

**932631 *Aseptis fanatica*–** The species name was misspelled as *fannatica* in Lafontaine & Schmidt (2010).

**932693.1 *Fishia nigrescens* –** Addition (see Crabo et al. 2013).

**932711.1 *Ufeus felsensteini* –** Addition (see Lafontaine and Walsh 2013).

**933606 *Xestia perquiritata orca* –** New subspecies (see Crabo et al. 2013).
